# *Polygonatum odoratum* Polysaccharides Modulate Gut Microbiota and Mitigate Experimentally Induced Obesity in Rats

**DOI:** 10.3390/ijms19113587

**Published:** 2018-11-13

**Authors:** Yan Wang, Yanquan Fei, Lirui Liu, Yunhua Xiao, Yilin Pang, Jinhe Kang, Zheng Wang

**Affiliations:** 1 College of Bioscience and Biotechnology, Hunan Agricultural University, Changsha 410128, China; 3087@huhst.edu.cn (Y.W.); FYQ0614@stu.hunau.edu.cn (Y.F.); Huazipiaoling.123@163.com (Y.X.); ylpang2010@126.com (Y.P.); 2 College of Agriculture and Biotechnology, Hunan University of Humanities, Science and Technology, Loudi 417000, China; 3 CAS Key Laboratory for Agro-Ecological Processes in Subtropical Region, National Engineering Laboratory for Pollution Control and Waste Utilization in Livestock and Poultry Production, South-Central Experimental Station of Animal Nutrition and Feed Science in Ministry of Agriculture, Institute of Subtropical Agriculture, The Chinese Academy of Sciences, Changsha 410125, China; 4 School of Biological Sciences, The University of Hong Kong, Pokfulam Rd, Hong Kong, China; liulirui0731@gmail.com

**Keywords:** *Polygonatum odoratum*, polysaccharides, high-fat diet, obesity, gut microbiota

## Abstract

Increasing evidence suggests that the gut microbiota plays vital roles in metabolic diseases. *Polygonatum odoratum* extract alleviates hyperglycemia and hyperlipidemia, but the underlying mechanism remains unclear. This study investigated the effects of *P. odoratum* polysaccharides (POPs) on high-fat diet (HFD)-induced obesity in rats and whether these effects were related to modulation of gut microbiota. POP treatment attenuated weight gain, fat accumulation, epididymal adipocyte size, liver triglycerides, and total liver cholesterol content in HFD-fed rats. POP administration also increased short-chain fatty acids (SCFAs), including isobutyric acid, butyric acid, and valeric acid. POP upregulated the expression of genes involved in adipocyte differentiation (*Pparg*, *Cebpa*, *Cebpb*) and lipolysis (*Ppara*, *Atgl*), and downregulated those related to lipid synthesis (*Srebpf1*, *Fabp4*, *Fas*), with corresponding changes in PPARγ and FABP4 protein expression. Finally, POP enhanced species richness and improved the gut microbiota community structure, reducing the relative abundances of *Clostridium*, *Enterococcus*, *Coprobacillus*, *Lactococcus*, and *Sutterella*. Principal coordinates analysis (PCoA) revealed a clear separation between HFD-fed rats and all other treatment groups. Correlation analysis identified negative and positive associations between obesity phenotypes and 28 POP-influenced operational taxonomic units (OTUs), including putative SCFA-producing bacteria. Our data suggest that POP supplementation may attenuate features of obesity in HFD-fed rats in association with the modulation of gut microbiota.

## 1. Introduction

Globally, obesity has increased at an alarming rate. In 2016, the World Health Organization found that 39% of adults worldwide were overweight, while 13% were obese [1]. This trend is a critical public health concern, given the link between obesity and chronic illnesses, including type II diabetes, cardiovascular disease, cancer, and fatty liver disease [2]. High energy intake and lack of physical activity are largely responsible for the dramatic increase in obesity rates. However, the development of obesity involves a host of complex interactions between genetics and the environment. Growing evidence has linked gut microbiota composition to changes in body weight and energy homeostasis [3,4]. Specifically, higher proportions of pathogenic *Sutterella*, *Desulfovibrionaceae*, *Streptococcaceae*, and *Clostridium* species are positively correlated with obesity development [5,6,7,8]. Furthermore, proportions of beneficial gut microbes (e.g., *Bifidobacterium*, *Lactobacillus*, *Akkermansia muciniphila*) decrease with weight gain associated with a high-fat diet (HFD) [8,9]. The anti-obesity effects of gut bacteria may be attributable to their generation of metabolites, such as short-chain fatty acids (SCFAs) and bile acids [10]. Both of these compounds have positive influences on defense-related processes, including immunity, inflammation, and lipid and glucose metabolism [3]. Thus, regulating intestinal microbiota dysbiosis is an effective method for preventing obesity and associated diseases [11,12].

Plant-based polysaccharide supplementation appears to protect against obesity-related metabolic syndrome [13], preventing weight gain, enhancing immunity, and improving gut microbiota composition in experimentally obese mice [11,14]. A promising source of polysaccharides is *Polygonatum odoratum* (Mill.) Druce, a traditional Chinese herb used as a preventative measure against diabetes, hyperlipidemia, and some cancers [15,16,17]. *P. odoratum* polysaccharides (POP) have been confirmed to be the primary contributor to the majority of observed benefits [18]. In experimentally induced obese mice, POP ameliorates metabolic disorders and enhances the mRNA expression of peroxisome proliferator-activated receptors (PPARs) [19], while preventing CCl_4_-induced liver oxidative injury [20]. Our previous in vitro study demonstrated that polysaccharides from *P. odoratum* rhizomes exerted antioxidant and antimicrobial activity against the pathogenic bacteria *Staphylococcus aureus*, *Pseudomonas aeruginosa*, *Bacillus subtilis*, and *Escherichia coli* [21]. Furthermore, *Polygonatum kingianum* polysaccharides significantly improve gut microbiota composition, decreasing the risk of type II diabetes [22]. Despite these promising findings, there is a lack of concrete data regarding whether POP modulates the gut microbiota in response to obesity. 

Thus, this study aimed to investigate whether POP treatment prevents the development of obesity and modulates gut microbiota dysbiosis in obese rats. More importantly, we aimed to identify potential correlations between POP treatment and changes in the gut microbiota, providing potential insight into gut microbiota-related mechanisms underlying the anti-obesity effects of POP.

## 2. Results

### 2.1. Response of Obese Rats to POP Treatment

**Figure 1:**
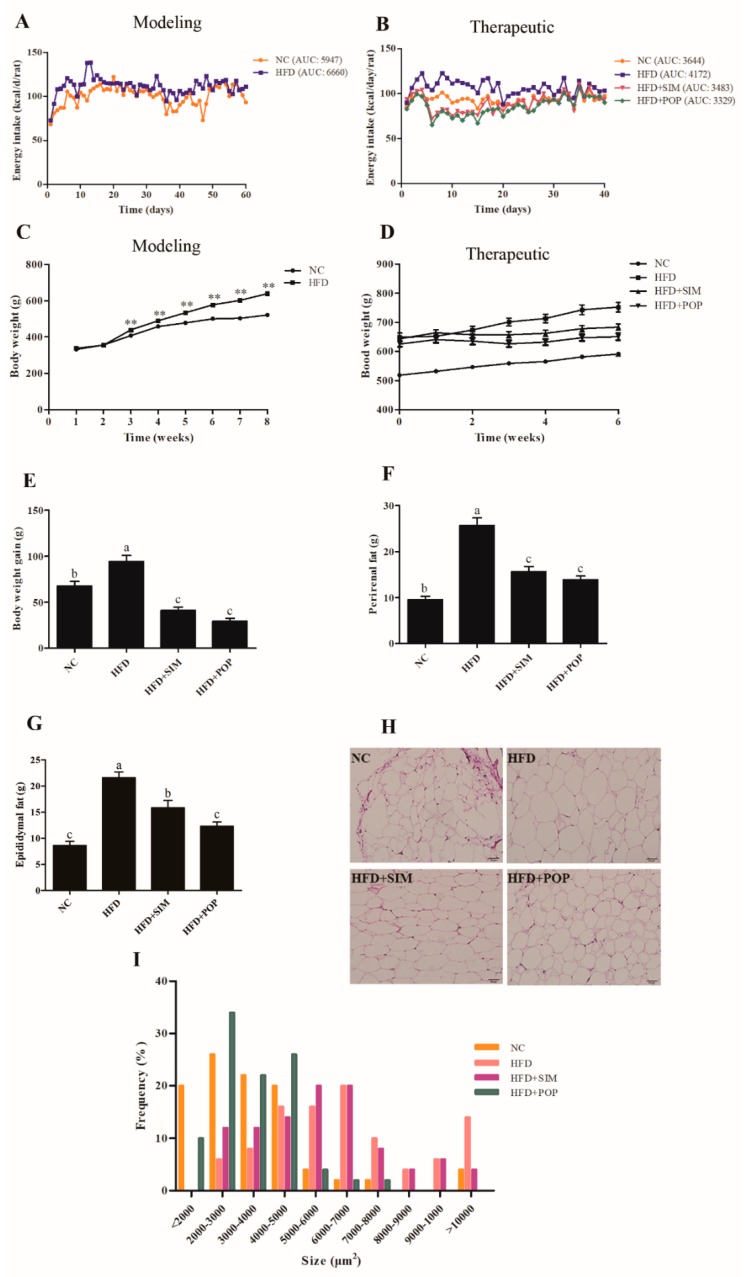
Effects of POP on HFD-induced obesity in rats. (**A**) Energy intake during modeling time. (**B**) Energy intake during treatment. (**C**) Body weight during modeling time. (**D**) Body weight during treatment. (**E**) Body weight gain during treatment. (**F**) Perirenal fat. (**G**) Epididymal fat. (**H**) Epididymal adipocyte microsections. (**I**) Epididymal adipocyte size evaluated by ImageJ software. NC = normal control, HFD = high-fat diet, HFD + SIM = HFD with simvastatin (1.8 mg/kg), HFD + POP = HFD with *P. odoratum* polysaccharides (400 mg/kg). AUC = Area under curve. All data are presented as means ± SEM. ** *p* < 0.01 for HFD (*n* = 30) versus NC (*n* = 10) in (**C**). ^a–c^ Mean values not sharing a common superscript were significantly different among the groups (*n* = 10 for each) (*p* < 0.05).

**Figure 2:**
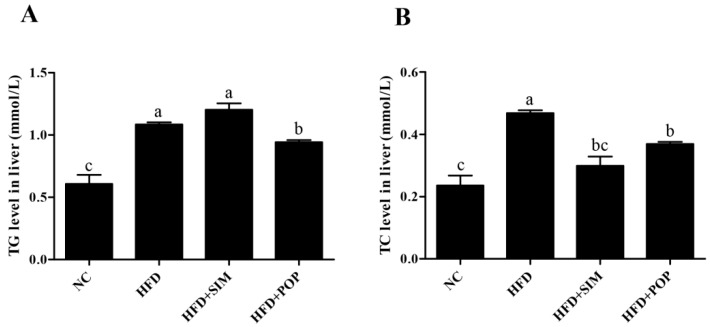
Effects of POP on TG (**A**) and TC (**B**) content in the livers of HFD-fed rats during the first six weeks. NC = normal control, HFD = high-fat diet, HFD + SIM = HFD with simvastatin (1.8 mg/kg), HFD + POP = HFD with *P. odoratum* polysaccharides (400 mg/kg). All data are presented as means ± SEM. ^a–c^ Mean values not sharing a common superscript were significantly different among the groups (*n* = 10 for each) (*p* < 0.05).

During establishment of the model (modeling time), total energy intake in the HFD group was higher than that in the normal control (NC) group (Figure 1A). In addition, during treatment, total energy intake in the HFD group was higher than those of all other groups [NC, HFD + simvastatin (SIM), HFD + POP] (Figure 1B). After eight weeks of HFD feeding, the average body weight of HFD rats was 22.63% higher than that of NC (Figure 1C). By week 14 (end of experiment), feeding of the HFD had significantly increased body weight, epididymal and perirenal fat accumulation, epididymal adipocyte size, and hepatic triglyceride (TG) and total cholesterol (TC) content (all *p* < 0.05; Figure 1D–I and Figure 2A,B). Notably, the POP and SIM treatments significantly decreased weight gain, body fat accumulation, epididymal adipocyte size, and hepatic TC content compared with those in the HFD-only group (all *p* < 0.05; Figure 1E–I and Figure 2B). Moreover, POP also significantly reduced liver TG content (*p* < 0.05; Figure 2A). Thus, POP appears to attenuate body weight gain and fat accumulation in rats with HFD-induced obesity by regulating total energy intake. 

### 2.2. Polysaccharide Treatment Altered the Expression of Lipid Metabolism-Related Genes in Adipose Tissue

**Figure 3:**
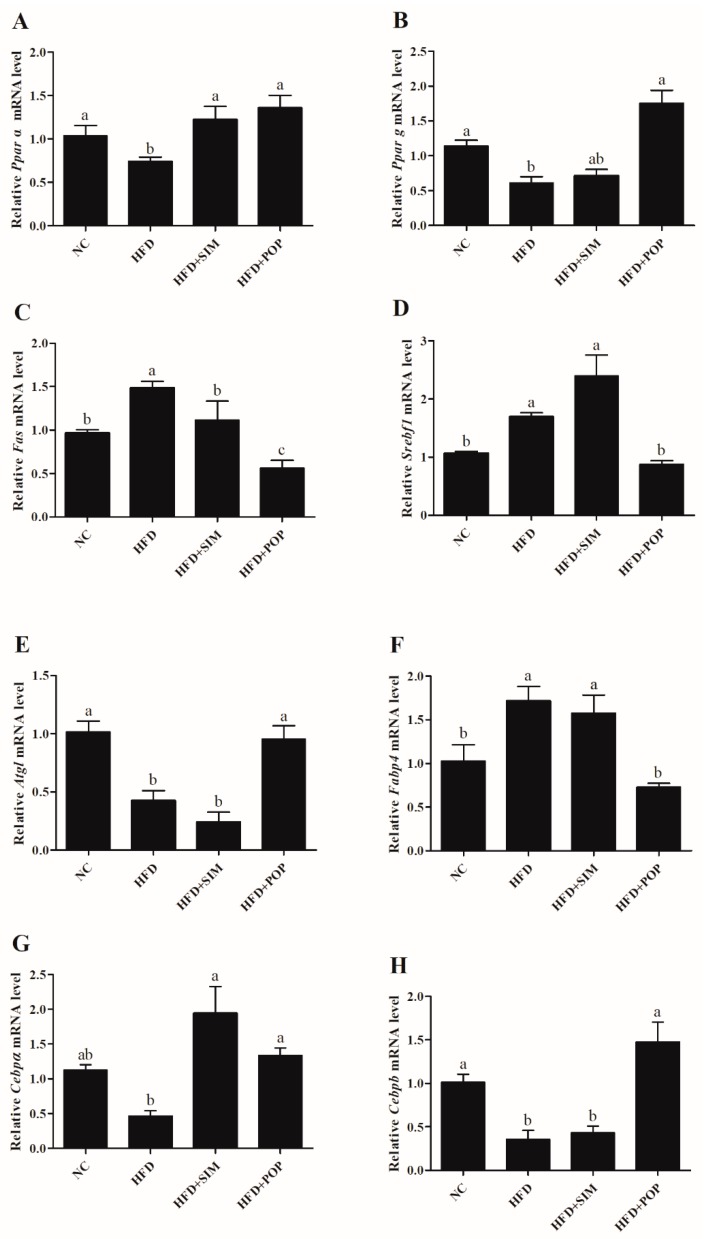
POP treatment modulated the mRNA expression of lipid metabolism-related genes in HFD-fed rats. Quantitative real-time PCR was used to assess (**A**) *Ppara*, (**B**) *Pparg*, (**C**) *Fas*, (**D**) *Srebf1*, (**E**) *Atgl*, (**F**) *Fabp4*, (**G**) *Cebpa*, and (**H**) *Cebpb* mRNA expression in adipose tissue. NC = normal control, HFD = high-fat diet, HFD + SIM = HFD with simvastatin (1.8 mg/kg), HFD + POP = HFD with *P. odoratum* polysaccharides (400 mg/kg). All data are presented as means ± SEM. ^a–c^ Mean values not sharing a common superscript were significantly different among the groups (*n* = 8 for each) (*p* < 0.05).

Compared with those in the NC group, HFD-fed rats exhibited upregulation of mRNAs encoding fatty acid synthase (Fas), sterol regulatory element-binding transcription factor 1 (Srebf1), and fatty acid binding protein 4 (Fabp4) (Figure 3C,D,F), along with downregulation of those encoding peroxisome proliferator-activated receptor α (Ppara), PPARγ (Pparg), adipose triglyceride lipase (Atgl), CCAAT/enhancer-binding protein α (Cebpa), and Cebpb (Figure 3A,B,E,G,H). When obese rats were treated with POP, however, expression of Fas, Srebf1, and Fabp4 significantly decreased (*p* < 0.05) (Figure 3C,D,F), while that of Ppara, Pparg, Atgl, Cebpa, and Cebpb increased (*p* < 0.05) (Figure 3A,B,E,G,H). In particular, the HFD + POP and NC groups did not significantly differ in expression of Ppara, Pparg, Atgl, Fabp4, Cebpa, and Cebpb (Figure 3A,B,E,F,G,H). Besides, the former group exhibited lower Fas mRNA expression (*p* < 0.05) (Figure 3C). 

**Figure 4:**
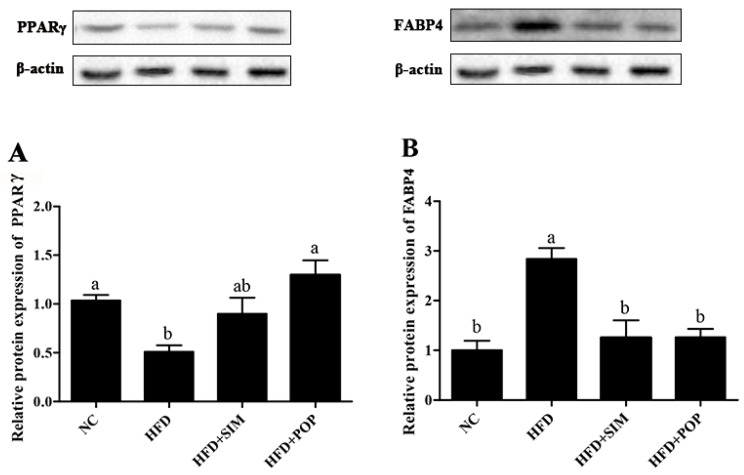
Western blotting analysis of PPARγ and FABP4 relative expression in HFD-fed rats. (**A**) PPARγ protein expression, (**B**) FABP4 protein expression. NC = normal control, HFD = high-fat diet, HFD + SIM = HFD with simvastatin (1.8 mg/kg), HFD + POP = HFD with *P*. *odoratum* polysaccharides (400 mg/kg). All data are presented as means ± SEM. ^a,b^ Mean values not sharing a common superscript were significantly different among the groups (*n* = 5 for each) (*p* < 0.05).

Treatment with SIM significantly decreased *Fas* mRNA expression (*p* < 0.05) and increased *Ppara* and *Cebpa* mRNA expression (both *p* < 0.05) compared with levels in the HFD group (Figure 3A,C,G). Similarly, POP significantly increased HFD-induced inhibition of PPARγ protein expression and attenuated FABP4 protein expression compared with those in the HFD group (Figure 4). Therefore, POP may reduce lipid accumulation by regulating the expression of genes involved in lipid metabolism in rats with HFD-induced obesity.

### 2.3. POP Induced Structural Changes in Gut Microbiota 

**Figure 5:**
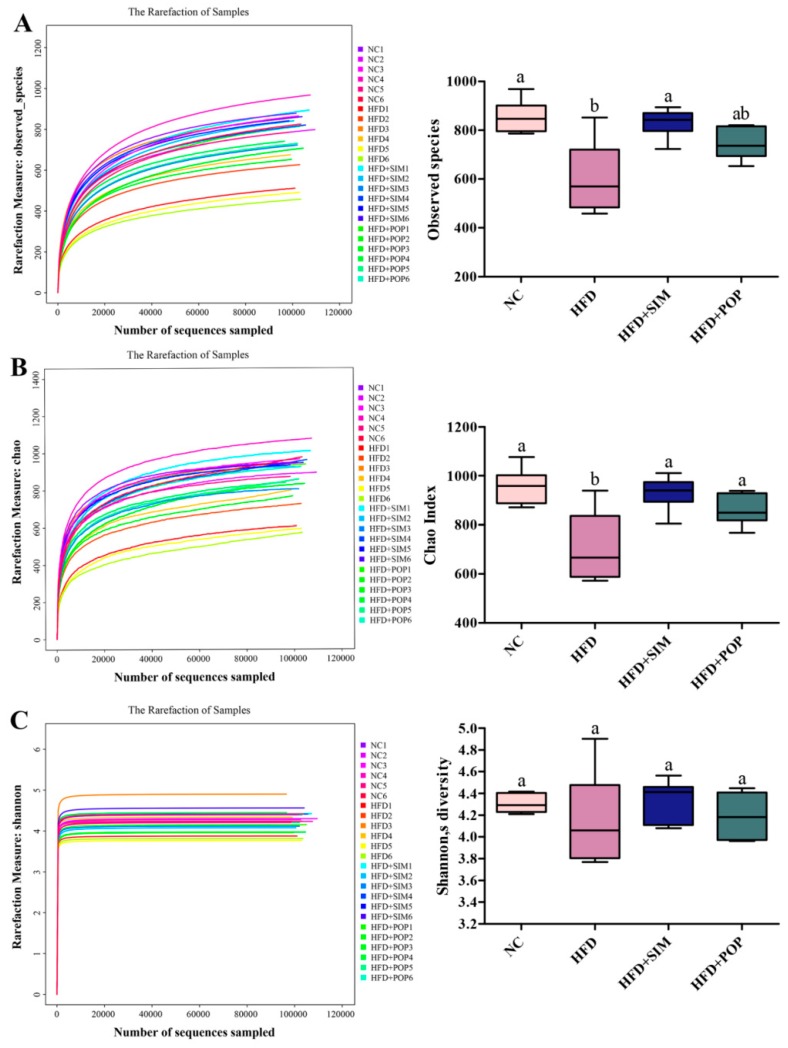
Diversity analysis of gut microbiota from fecal samples of POP-treated obese rats. (**A**) Observed species. (**B**) Chao index. (**C**) Shannon’s diversity. NC = normal control, HFD = high-fat diet, HFD + SIM = HFD with simvastatin (1.8 mg/kg), HFD + POP = HFD with *P*. *odoratum* polysaccharides (400 mg/kg). All data are presented as means ± SEM. ^a,b^ Mean values not sharing a common superscript were significantly different among the groups (*n* = 6 for each) (*p* < 0.05).

After removing low-quality sequences, we obtained 10,490,150 raw reads (24 samples, *n* = 6 for each group), with an average of 437,089 ± 14,795 reads per sample. We then generated 9,355,208 clean reads, averaging 748,416 ± 13,009 per fecal sample. Rarefaction analysis suggested that the current sequencing depth successfully captured rare and new phylotypes, as well as the majority of gut microbial diversity (Figure 5). The HFD group exhibited significantly fewer observed species and a significantly lower Chao index (i.e., species richness) (*p* < 0.05) compared to those in the NC group (Figure 5A,B). However, the Shannon index (species diversity) in the HFD group was lower than that in NC rats, although this difference was not statistically significant (*p* > 0.05, Figure 5C). Under POP treatment, the Chao index was significantly higher than that in the HFD group (*p* < 0.05), while both the Shannon index and observed species number tended to be higher as well (*p* = 0.07 and 0.08 respectively). These results support the view that POP ameliorates decrease in gut microbiota richness stemming from obesity. 

**Figure 6:**
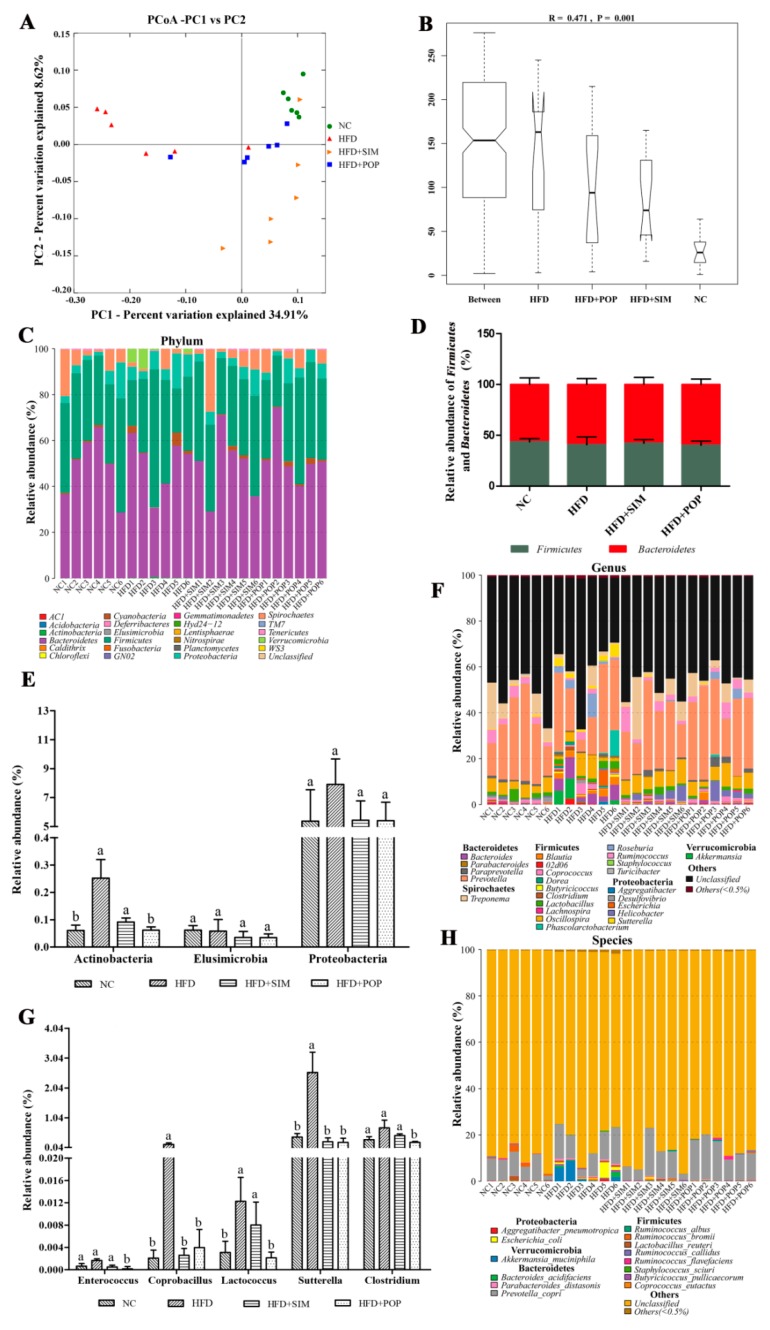
POP treatment regulated gut microbiota structure in HFD-fed obese rats. (**A**) UniFrac-based PCoA plots. (**B**) Analysis of similarity (ANOSIM) among groups based on the Bray–Curtis distances. (**C**) Relative abundances of gut microbiota at the phylum level. Relative abundances of (**D**) major phyla *Firmicutes* and *Bacteroidetes* and (**E**) minor phyla *Actinobacteria*, *Elusimicrobia*, and *Proteobacteria*. Relative abundances of gut microbiota at the (**F**) genus and (**H**) species levels. (**G**) Relative abundances of minor genera *Enterococcus*, *Coprobacillus*, *Lactococcus*, *Sutterella*, and *Clostridium*. All data are presented as means ± SEM. ^a,b^ Mean values not sharing a common superscript were significantly different among the groups (*n* = 6 for each) (*p* < 0.05).

UniFrac-based principal coordinates analysis (PCoA) results revealed a distinct clustering of the gut microbiota composition for each treatment group, with the HFD group clearly separated from the remaining three groups (Figure 6A). As expected, POP treatment increased the similarity between the overall gut microbiota compositions of the HFD + POP and NC groups, suggesting that POP had substantial effects on the gut microbiota composition of HFD-fed rats. Similarity analysis (ANOSIM, Figure 6B) showed that the distances between groups were significantly larger than those within groups (*R* = 0.471 and *p* = 0.001).

Taxonomic analysis suggested that the fecal microbiota was represented by four major phyla: *Firmicutes*, *Bacteroidetes*, *Proteobacteria*, and *Spirochaetes* (Figure 6C). Neither *Firmicutes* (F) and *Bacteroidetes* (B) abundances, nor the F/B ratio, significantly differed among the four treatment groups (Figure 6D). In contrast, HFD feeding significantly increased the relative abundance of *Actinobacteria*, compared to that in the NC group (*p* < 0.05; Figure 6E). However, POP treatment significantly reversed this HFD-induced increase (*p* < 0.05) and tended to have an inhibitory effect on the relative abundance of *Proteobacteria* (*p* = 0.08; Figure 6E). 

At the genus level, HFD significantly enhanced the relative abundances of *Coprobacillus*, *Lactococcus*, and *Sutterella* (all *p* < 0.05; Figure 6F,G). The relative abundances of *Coprobacillus* and *Sutterella* were restored to NC levels upon POP and SIM treatment (both *p* < 0.05). Likewise, *Clostridium*, *Enterococcus*, and *Lactococcus* relative abundances also decreased in the HFD + POP group compared with those in the HFD group (*p* < 0.05; Figure 6F,G).

### 2.4. Key Phenotypes Responding to POP Treatment of HFD-Fed Rats

**Figure 7:**
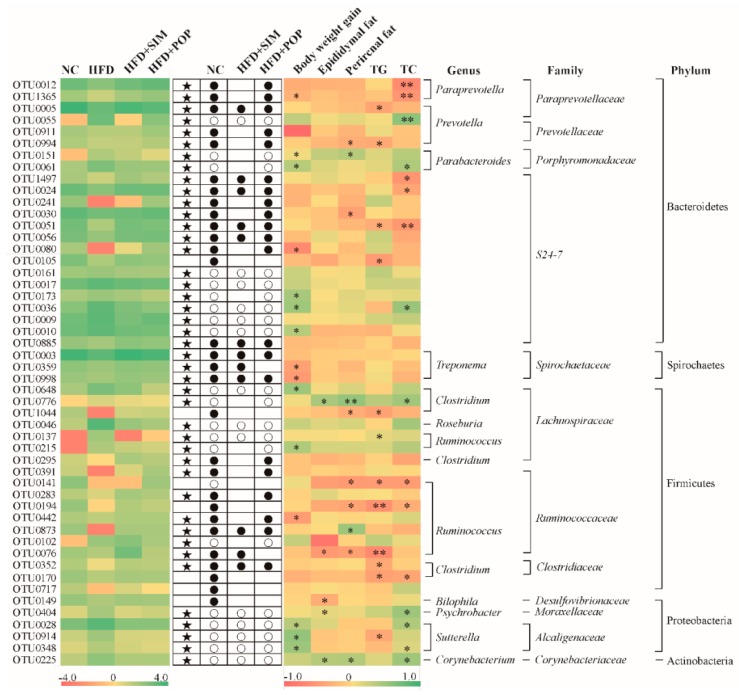
Heat map of RDA-identified key OTUs in response to POP treatment according to Spearman’s correlations between the identified OTUs and weight gain, epididymal fat, perirenal fat, and liver TG and TC levels. Colors of squares on the left indicate the average abundance of the OTU in each group. Open (○) and closed (●) circles represent lower and higher relative abundances (log_10_ transformed), respectively, of OTUs in NC, HFD + SIM, or HFD + POP groups compared with that in the HFD group. Stars (★) indicate OTUs in the NC group altered by HFD intervention that were restored by POP or SIM treatment. Colors of squares on the right indicate *R*-values of Spearman’s correlation between OTU and weight gain, epididymal fat, perirenal fat, and liver TG and TC levels. Phylum, family, and genus names of the OTUs are shown on the right. * *p* < 0.05 and ** *p* < 0.01.

We identified 5124, 3617, 4984, and 4464 operational taxonomic units (OTUs) in the NC, HFD, HFD + SIM, and HFD + POP groups, respectively. HFD treatment significantly altered the abundances of 50 OTUs, among which 22 were enriched while the other 28 were reduced compared with those in the NC group (Figure 7, Table A1 and Table A2). Additionally, POP and SIM treatments significantly changed the abundance of 41 (21 increased and 20 decreased) and 26 (12 increased and 14 decreased) OTUs (Figure 7, Table A2) in comparison with those in the HFD group, respectively.

Spearman’s correlation analysis identified 28 key variables that were significantly altered after POP treatment and negatively or positively correlated with alterations in body weight gain, epididymal fat, perirenal fat, and hepatic TG and TC levels (Figure 7). Among them, five families were negatively correlated with obesity parameters: *Paraprevotellaceae* (OTU0012, OTU1365, and OTU0005 from *Bacteroidetes*), *Prevotellaceae* (OTU0994 from *Bacteroidetes*), S24-7 (OTU1497, OTU0024, OTU0030, OTU0051, and OTU0080 from *Bacteroidetes*), *Spirochaetaceae* (OTU0359 and OTU0998 from *Spirochaetes*), and *Ruminococcaceae* (OTU0442 from *Firmicutes*). In contrast, seven families were positively correlated with obesity: *Paraprevotellaceae* (OTU0055), *Porphyromonadacea* (OTU0151 and OTU0061), S24-7 (OTU0173, OTU0036, and OTU0010), *Lachnospiraceae* (OTU0648, OTU0776, OTU0137, and OTU0215), *Ruminococcaceae* (OTU0873), *Moraxellaceae* (OTU0404), *Alcaligenaceae* (OUT0028, OTU0914, and OTU0348), and *Corynebacteriaceae* (OTU0225). Taken together, the results demonstrate that POP treatment modulates HFD-induced gut microbiota dysbiosis, resulting in the restoration of a microbiota community similar to that in controls.

### 2.5. POP Regulated SCFAs in Feces

Apart from a reduction in total SCFAs in the HFD + SIM group, the remaining groups (NC, HFD, HFD + POP) did not significantly differ in SCFA levels (Table 1). When comparing the HFD group to the NC group, however, propionic acid, butyrate acid, and valeric acid concentrations in the former were reduced by 18.41%, 77.15%, and 27.84%, respectively. Furthermore, POP treatment increased concentrations of isobutyric acid (by 47.03%), butyrate acid (587.41%), and valeric acid (93.42%) compared to those in the HFD group. In contrast, the HFD and HFD + SIM groups did not significantly differ in the production of butyric acid or valeric acid. 

## 3. Discussion

In this study, we found that POP supplementation ameliorated negative effects of obesity, including weight gain, increased adipocyte size, and elevated liver TG/TC levels, in line with several previous studies [17,23]. 

Dietary polysaccharides generally escape digestion in the stomach and the small intestine, reaching the large intestine, where they are available for fermentation and thereby enhance the growth of beneficial bacteria and improve intestinal microbial diversity [24]. In addition, the gut microbiota may regulate energy harvest, which is related to lipid metabolism and the development of obesity [25]. Furthermore, numerous reports have indicated that dietary polysaccharides from plants reduce physiological risk factors (e.g., obesity, hyperlipidemia, and inflammation) by modulating the gut microbiota [11,13,26]. Therefore, we speculated that the inhibitory effect of POP on body weight gain and fat accumulation in our study may be owing to the modification of the HFD-fed obese rat gut microflora. We found that while POP had no significant effect on the relative abundances of *Firmicutes* (F) or *Bacteroides* (B), the F/B ratio, or species diversity (Shannon’s diversity), POP supplementation enhanced overall species richness (Chao index). Notably, there was also no significant difference in the *Firmicutes* and *Bacteroides* relative abundances or species diversity between the HFD and NC groups. This result is similar to the findings of Zhen et al. [14], who reported that Fuzhuan brick-tea polysaccharides increased species richness but had limited effects on the relative abundances of *Firmicutes* and *Bacteroides*. Shannon’s diversity is an indicator of species evenness in samples [27]. Although POP significantly increased the abundances of certain taxa, the species evenness of the HFD + POP group was not increased, as no significant difference was observed in Shannon’s index between the HFD and HFD + POP groups. Moreover, POP treatment reduced levels of some *Firmicutes* subgroups but not others, while only one *Bacteroidetes* subgroup experienced a decline. Thus, supplementation-related weight loss may affect these subgroups differently [28]. We also observed a significant decrease in *Actinobacteria* abundance after POP supplementation, suggesting an anti-obesity effect, as this bacterial phylum has been linked to obesity [29,30]. Likewise, POP tended to reduce levels of *Proteobacteria*, a major phylum of pathogenic gram-negative bacteria [31,32]. Our results showed that POP intake also reduced the abundance of *Sutterella*, corresponding to a previous report by Tang et al. showing that polysaccharides derived from purple sweet potato reduced the abundance of *Sutterella* (a member of the *Proteobacteria*) compared with that in cyclophosphamide-treated mice [33]. 

Our correlation analyses revealed 28 key OTUs that were significantly associated with at least one obesity parameter. Treatment with POP significantly enhanced the relative abundances of 12 OTUs that may represent beneficial gut flora. These 12 OTUs were negatively correlated with obesity phenotypes and produce SCFAs through fermenting undigested polysaccharides [34,35]. Several of these OTUs correspond to bacteria that have previously been associated with obesity-mitigating effects, such as *Lachnospiraceae*, *S24-7*, and *Ruminococcaceae* [14,22]. 

Treatment with POP also significantly reduced the proportions of 16 OTUs that were positively correlated with obesity phenotypes. Four of these genera (*Corynebacterium, Clostridium*, *Parabacteroides*, and *Sutterella*) have been implicated in obesity-related metabolic disorders [36,37,38]. Furthermore, OTU_0225 and OTU_0776 were significantly correlated with epididymal fat, perirenal fat, and liver TC, indicating that they may be central to the mechanism by which POP ameliorates obesity. Gene sequencing data revealed that OTU_0776 corresponds to *Clostridium aldenense*, a common pathogen [39]. The present results further illustrate that the anti-obesity activity of POP may be associated with modulation of the gut microbiota.

Polysaccharides are not digestible by gastrointestinal digestive enzymes but can be fermented by gut microbiota. This process produces beneficial metabolites, most notably SCFAs (acetate, propionate, butyrate, and valerate). High SCFA levels are unfavorable to several potentially pathogenic bacteria [40] but promote the proliferation of *Bacteroides*, *Ruminococcus*, *Lactobacillus*, *Prevotella*, and *Butyricicoccus* [41,42]. Our results showed that POP upregulated isobutyric acid, butyric acid, and valeric acid in HFD + POP-fed rats, resulting in changes to *Ruminococcus* and *Prevotella* abundance. Similar findings have been reported for seaweed polysaccharides, which enhance SCFA production by stimulating the growth of beneficial microbes (*Ruminococcaceae*, *Bacteroidetes*, and *Lactobacillus*) [43]. These results indicate that POP enhances the growth of SCFA-producing bacteria in the gut, such as *Ruminococcus* and *Prevotella*. 

The present study revealed that POP supplementation reduced the expression of *Fas*, *Srebf1*, and *Fabp4* in adipose tissues of HFD-fed rats, while increasing the expression of *Ppara*, *Pparg*, *Atgl*, *Cebpa*, and *Cebpb*. All of the corresponding proteins are involved in lipid metabolism. PPARα modulates the transcription of genes involved in fatty acid oxidation and energy expenditure [44], as well as increasing fatty acid combustion, which leads to a reduction in hepatic TG content [45]. Moreover, SREBP-1c activates the transcription of genes involved in fatty acid and TG synthesis, such as *Fas* [46]. PPARγ plays an important role in maintaining lipid homeostasis by controlling the expression of genes functioning in lipid and carbohydrate metabolism [47,48]. PPARγ activation may promote adipocyte differentiation and fat accumulation [49,50,51] but also promotes fatty acid oxidation; increases small, relatively insulin-sensitive adipocytes [44]; and attenuates inflammation [52,53]. Our findings are supported by previous studies demonstrating that *Rhizoma Polygonati Odorati* extracts from the root of *P. odoratum* upregulate PPARγ expression and downregulate FABP4 expression in the adipocyte tissues of HFD-fed obese rats [19]. In contrast, other studies have shown that HFD feeding increases *Pparg* expression, whereas resveratrol and germacrone decrease *Pparg* mRNA expression compared with that in HFD-fed mice [54,55]. These inconsistent results may be partially due to differences in treatment conditions, diet ingredients, experimental duration, and species. Moreover, because SCFAs are PPARγ modulators [36], dietary changes may alter SCFA profiles and, in turn, PPARγ signaling. Previous research has indicated that butyrate supplementation upregulates PPARγ expression in pig adipocytes [56] and prevents diet-induced obesity by increasing energy expenditures [57]. Therefore, our results suggest that POP may induce butyrate production and thus alter *Pparg*, *Ppara*, and adipogenesis-related gene expression. As a result, obesity-related symptoms are suppressed.

## 4. Materials and Methods

### 4.1. Animals

Forty male Sprague–Dawley (SD) rats (average weight, 280 ± 20 g, 8 weeks old) with certificate no. HNASLKJ2016-0002 were purchased from Hunan Sileike Jingda Co. (Changsha, China). Subjects were housed in plastic-bottomed cages with reticulate stainless-steel covers under controlled light conditions (12-h light-dark cycle). The study was approved by the Animal Care Committee on the Ethics of Animal Experiments of Institute of Subtropical Agriculture (15 May 2017, Changsha, China). All animal protocols were in accordance with the Regulation on Management of Experimental Animals (Hunan Province, No. 259, 2012).

### 4.2. Animal Diets

Subjects had ad libitum access to food and water. The control chow diet (total energy content: 3.6 kcal/g) consisted of 72.3%, 19.7%, and 10% of kilocalories from carbohydrates, protein, and fat, respectively. The HFD (total energy content: 4.6 kcal/g) contained 46.4%, 19.6%, and 34.0% of kilocalories from carbohydrates, proteins, and fat, respectively. Chow was ordered from Botai Hongda Biotechnology Co. (Beijing, China).

### 4.3. Reagents

POP of 98% purity was purchased from Shaanxi Ciyuan Biotechnology Co. (Xi’an, China). SIM from Mo Sha Dong (Hangzhou, China) was used as the reference hypolipidemic drug. High-purity SCFA standards—including acetic acid, propionic acid, isobutyric acid, butyric acid, isovaleric acid, and valeric acid—were purchased from Sigma Aldrich Chemical, Co. (St. Louis, MO, USA). All other chemicals used were of analytical grade.

### 4.4. Experimental Design

After one week of acclimatization, rats were randomly assigned to two groups fed different chows: (1) NC (*n* = 10) and (2) HFD (*n* = 30). The HFD rats were considered obese after eight weeks (HFD rats exhibited 20% higher body weight than NC rats [58]) and were randomly divided into three groups of 10 rats each: (1) HFD-only; (2) HFD + SIM, fed an HFD and intragastrically administered 1.8 mg/kg body weight SIM once daily [59]; and (3) HFD + POP, fed an HFD and intragastrically administered 400 mg/kg body weight POP once daily [60]. From week 9, the NC and HFD groups were given 400 mg/kg sterile saline intragastrically. 

**Figure 8:**
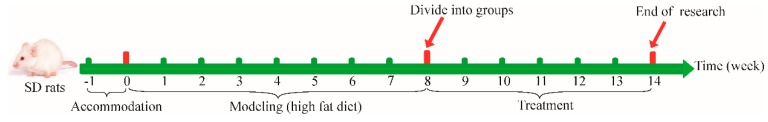
A schematic diagram of the experimental treatment schedule. All rats except normal control (NC) rats were raised on an HFD for 8 weeks. Obese rats (body weight > 20% higher than in NC rats) were then divided into three groups: HFD (high-fat diet), HFD + SIM (high-fat diet with simvastatin), and HFD + POP (high-fat diet with *Polygonatum odoratum* polysaccharides). All rats were gavage-dosed once per day for six consecutive weeks (weeks 8–14).

During the experiment, rats were weighed once weekly. The experiment ended on week 14, when fecal samples of all rats were collected, immediately transferred to pre-labeled microcentrifuge tubes, and frozen in liquid nitrogen until further processing. Subsequently, after 12 h of food deprivation, all animals were anesthetized with pentobarbital sodium (35 mg/kg) and euthanized through cervical dislocation. Perirenal fat and epididymal fat were excised, weighed, and immediately frozen in liquid nitrogen. Figure 8 provides a schematic of the treatment schedule. 

### 4.5. Biochemical Analysis

TG and TC contents in liver were measured with a BS-200 full automatic biochemical analyzer (Shenzhen Mindray Co., Shenzhen, China) using corresponding reagent kits and following published protocols [61].

### 4.6. SCFA Analysis in Feces

Fecal SCFAs were measured using gas chromatography, following a previously described method [61], with some modification. Fresh fecal samples (0.5 g) were diluted in 1 mL of 10% perchloric acid. After shaking for 30 min, the mixture was centrifuged multiple times at 12,000× *g* and 4 °C for 15 min, until the supernatant was clear. After filtering through syringe filters (45 µm diameter), the supernatant was injected into an Agilent 6890 N gas chromatography equipped with a flame ionization detector (FID), high-resolution gas chromatography column (DB-FFAP, 30 m × 0.25 mm × 0.25 μm), and N10149 automatic liquid sampler (Agilent, Santa Clara, CA, USA). The amount of acetic acid, propionic aid, isobutyric acid, butyric acid, isovaleric acid, and valeric acid in each sample was quantified using external calibration curves.

### 4.7. RNA Extraction and Quantitative Real-Time Polymerase Chain Reaction (qRT-PCR)

Total RNA from adipose tissues was extracted using TRIzol reagent (Invitrogen, Carlsbad, CA, USA). An ND-1000 UV-vis spectrophotometer (NanoDrop Ltd., Waltham, MA, USA) was then used to evaluate RNA concentration and integrity. First-strand cDNA synthesis from RNA was performed using a PrimeScript^TM^ RT reagent kit (TaKaRa, Kyoto, Japan). Next, qRT-PCR was performed in triplicate using SYBR^®^ Premix EX Taq (TaKaRa), following the manufacturer’s protocol. Relative quantification was calculated using the 2^−∆∆Ct^ method. Primer sequences (Table A3) targeted the following rat genes: *Ppara*, *Pparg*, *Fas*, *Srebf1*, *Atgl*, *Fabp4*, *Cebpa*, and *Cebpb*.

### 4.8. Western Blotting

Adipose tissues (100 mg) were homogenized in 1 mL RIPA buffer containing the protease inhibitor cocktail Complete EDTA free (Roche, Penzberg, Germany) and phosphatase inhibitor Cocktail PhosSTOP (Roche). Protein concentration was determined with an enhanced BCA protein assay kit (P0010, Beyotime Biotechnology, Shanghai, China). Samples were denatured at 100 °C for 5 min and stored at −80 °C until needed for western blotting. To detect target proteins, the following primary antibodies were used: PPARγ (sc-271392, Santa Cruz Biotechnology, Santa Cruz, CA, USA, diluted 1:200), FABP4 (sc-374588, Santa Cruz Biotechnology, diluted 1:200), and β-actin (60008-1-Ig, Proteintech, Chicago, IL, USA, diluted 1:10,000). PPARγ and FABP4 protein levels in experimental groups are presented as fold changes relative to the average NC value.

### 4.9. Histology Analysis

Epididymal fat tissues isolated from rats in the four experimental groups were washed with saline, fixed in 4% paraformaldehyde, embedded in paraffin (SVA, Uppsala, Sweden), and sectioned (5 μm). Sections were then stained with hematoxylin and eosin for observation under an optical microscope. ImageJ (National Institutes of Health, Bethesda, MD, USA) was used to estimate adipose cell size. 

### 4.10. Gut Microbiota Analysis

Total DNA was isolated from fecal samples using a DNA Stool Mini Kit (Qiagen, Hilden, Germany) following the manufacturer’s protocol. The 515F-806R primer set was used to amplify the 16S rRNA gene containing the V4 variable region. The reaction mixture (50 µL) contained 25 µL NEB Phusion High-Fidelity PCR Master Mix (New England Biolabs, Ipswich, MA, USA), 0.5 µM of each primer, and 30 ng of template. Thermocycling procedures were as follows: pre-denaturation at 98 °C for 3 min; followed by 30 cycles of 98 °C for 45 s, 55 °C for 45 s, and 72 °C for 45 s; and a final extension for 7 min at 72 °C. Amplicons were purified with AmpureXPbeads (Agencourt, Beckman Coulter, Brea, CA, USA). The final library was quantified using the Agilent 2100 Bioanalyzer (Agilent, Palo Alto, CA, USA), then sequenced on an Illumina HiSeq 2500 to generate 250-bp paired-end reads. The raw reads were quality-filtered and merged using the following criteria [62]: (1) Raw reads were truncated at any site that gave rise to an average quality score <20. (2) Reads contaminated by adapter, ambiguous bases (N base) and more than 10bp bases of homopolymer were removed by BGI’s internal process. The remaining reads are overlapped to tags by Fast Length Adjustment of Short reads (FLASH, v1.2.11) (Baltimore, MD, USA), which were clustered into OTUs based on Ribosomal Database Project (RDP) [63] by UPARSE (Tiburon, CA, USA) with a 97% similarity cutoff [64]. Alpha diversity (Shannon’s and Simpson’s indexes), richness (Ace, observed species, and Chao1), and rarefaction curve analyses were performed by Mothur (v1.31.2) (Ann Arbor, MI, USA). UniFrac-based PCoA was performed by QIIME (v1.8.0) (Boulder, CO, USA). 

### 4.11. Statistical Analysis 

Unless otherwise stated, all data are presented as means ± SEM. The statistical significance of differences between groups was evaluated by one-way analysis of variance (ANOVA) followed by Tukey’s test using SPSS 21 software (IBM, Armonk, NY, USA). The statistical significance of the separation among treatment groups in the PCoA score plots was assessed using multivariate analysis of variance (MANOVA) according to UniFrac matrix differences. Differences in the relative abundances of OTUs were assessed using the Mann–Whitney test by the R package. (v3.1.1) (University of Auckland, Auckland, New Zealand). Spearman’s correlation analysis was used to determine the relationship between OTUs and obesity parameters. Furthermore, adjusted *p*-values obtained based on the false discovery rate (FDR) were used to evaluate differences in differential abundance. A value of *p* < 0.05 was considered to be significant in this work.

### 4.12. Accession Number 

The sequences have been deposited in the NCBI Sequence Read Archive Database under accession number SUB4662722.

## 5. Conclusions

In conclusion, POP supplementation attenuated body weight gain, epididymal and perirenal fat accumulation, and liver TG and TC levels in HFD-fed obese rats. These effects were associated with the regulation of lipid metabolism-related gene expression in adipocytes. Furthermore, structural alterations of the gut microbiota induced by HFD treatment were modulated by POP and may be associated with the POP’s anti-obesity activity. The present study further identified 28 key OTUs that were negatively or positively associated with obesity parameters and that may play a key role in preventing the occurrence and development of obesity. Taken together, our results demonstrate that the anti-obesity effects of POP may be related to modulation of the gut microbiota.

## Figures and Tables

**Table 1:** Effects of POP on SCFA production in HFD-fed obese rats.

SCFA (mg/g)	NC	HFD	HFD + SIM	HFD + POP
Acetic acid	1.29 ± 0.185a	1.01 ± 0.134ab	0.79 ± 0.069b	0.65 ± 0.156b
Propionic acid	0.32 ± 0.024a	0.26 ± 0.061b	0.13 ± 0.012bc	0.27 ± 0.081b
Isobutyric acid	0.018 ± 0.002ab	0.015 ± 0.002b	0.0078 ± 0.0004c	0.022 ± 0.003a
Butyric acid	0.88 ± 0.088b	0.199 ± 0.049c	0.29 ± 0.034c	1.37 ± 0.196a
Isovaleric acid	0.022 ± 0.001a	0.025 ± 0.003a	0.0097 ± 0.001b	0.028 ± 0.006a
Valeric acid	0.032 ± 0.004a	0.024 ± 0.003b	0.013 ± 0.001b	0.046 ± 0.009a
Total	2.21 ± 0.169a	2.26 ± 0.402a	1.17 ± 0.075b	2.9 ± 0.548a

All data are presented as means ± SEM. ^a–c^ Mean values not sharing a common superscript were significantly different among the groups (*n* = 6 for each) (*p* < 0.05).

## Abbreviations

POP	*Polygonatum odoratum* polysaccharides
SD	Sprague–Dawley
HFD	high-fat diet
NC	normal control
SIM	simvastatin
TG	triglyceride
TC	total cholesterol
SCFA	short-chain fatty acid
qRT-PCR	quantitative real-time polymerase chain reaction
Ppara	peroxisome proliferator-activated receptor α
Pparg	peroxisome proliferator-activated receptor γ
Fas	fatty acid synthase
Srebf1	sterol regulatory element-binding transcription factor 1
Atgl	adipose triglyceride lipase
Fabp4	fatty acid binding protein 4
Cebpa	CCAAT/enhancer-binding protein alpha
Cebpb	CCAAT/enhancer-binding protein beta
OTUs	operational taxonomic units
PCoA	principal coordinates analysis
S	supplemental

## Appendix A

**Table A1:** Fifty key OTUs responding to POP treatment, as identified by redundancy analysis.

OTU ID	Phylum	Class	Order	Family	Genus
**OTU0012**	*Bacteroidetes*	*Bacteroidia*	*Bacteroidales*	*Paraprevotellaceae*	*Paraprevotella*
**OTU1365**	*Bacteroidetes*	*Bacteroidia*	*Bacteroidales*	*Paraprevotellaceae*	*Paraprevotella*
**OTU0005**	*Bacteroidetes*	*Bacteroidia*	*Bacteroidales*	*Paraprevotellaceae*	*Prevotella*
**OTU0911**	*Bacteroidetes*	*Bacteroidia*	*Bacteroidales*	*Prevotellaceae*	*Prevotella*
**OTU0994**	*Bacteroidetes*	*Bacteroidia*	*Bacteroidales*	*Prevotellaceae*	*Prevotella*
**OTU1497**	*Bacteroidetes*	*Bacteroidia*	*Bacteroidales*	*S24-7*	
**OTU0024**	*Bacteroidetes*	*Bacteroidia*	*Bacteroidales*	*S24-7*	
**OTU0241**	*Bacteroidetes*	*Bacteroidia*	*Bacteroidales*	*S24-7*	
**OTU0030**	*Bacteroidetes*	*Bacteroidia*	*Bacteroidales*	*S24-7*	
**OTU0051**	*Bacteroidetes*	*Bacteroidia*	*Bacteroidales*	*S24-7*	
**OTU0056**	*Bacteroidetes*	*Bacteroidia*	*Bacteroidales*	*S24-7*	
**OTU0080**	*Bacteroidetes*	*Bacteroidia*	*Bacteroidales*	*S24-7*	
**OTU0105**	*Bacteroidetes*	*Bacteroidia*	*Bacteroidales*	*S24-7*	
**OTU0170**	*Firmicutes*	*Clostridia*	*Clostridiales*	*Clostridiaceae*	
**OTU0003**	*Spirochaetes*	*Spirochaetes*	*Spirochaetales*	*Spirochaetaceae*	*Treponema*
**OTU0359**	*Spirochaetes*	*Spirochaetes*	*Spirochaetales*	*Spirochaetaceae*	*Treponema*
**OTU0998**	*Spirochaetes*	*Spirochaetes*	*Spirochaetales*	*Spirochaetaceae*	*Treponema*
**OTU1044**	*Firmicutes*	*Clostridia*	*Clostridiales*	*Lachnospiraceae*	*Clostridium*
**OTU0194**	*Firmicutes*	*Clostridia*	*Clostridiales*	*Ruminococcaceae*	*Ruminococcus*
**OTU0283**	*Firmicutes*	*Clostridia*	*Clostridiales*	*Ruminococcaceae*	*Ruminococcus*
**OTU0295**	*Firmicutes*	*Clostridia*	*Clostridiales*	*Ruminococcaceae*	*Clostridium*
**OTU0391**	*Firmicutes*	*Clostridia*	*Clostridiales*	*Ruminococcaceae*	
**OTU0442**	*Firmicutes*	*Clostridia*	*Clostridiales*	*Ruminococcaceae*	*Ruminococcus*
**OTU0873**	*Firmicutes*	*Clostridia*	*Clostridiales*	*Ruminococcaceae*	*Ruminococcus*
**OTU0885**	*Bacteroidetes*	*Bacteroidia*	*Bacteroidales*	*S24-7*	
**OTU0076**	*Firmicutes*	*Clostridia*	*Clostridiales*	*Ruminococcaceae*	*Ruminococcus*
**OTU0352**	*Firmicutes*	*Clostridia*	*Clostridiales*	*Clostridiaceae*	*Clostridium*
**OTU0717**	*Firmicutes*	*Clostridia*	*Clostridiales*		
**OTU0149**	*Proteobacteria*	*Deltaproteobacteria*	*Desulfovibrionales*	*Desulfovibrionaceae*	*Bilophila*
**OTU0151**	*Bacteroidetes*	*Bacteroidia*	*Bacteroidales*	*Porphyromonadaceae*	*Parabacteroides*
**OTU0061**	*Bacteroidetes*	*Bacteroidia*	*Bacteroidales*	*Porphyromonadaceae*	*Parabacteroides*
**OTU0055**	*Bacteroidetes*	*Bacteroidia*	*Bacteroidales*	*Paraprevotellaceae*	*Prevotella*
**OTU0161**	*Bacteroidetes*	*Bacteroidia*	*Bacteroidales*	*S24-7*	
**OTU0017**	*Bacteroidetes*	*Bacteroidia*	*Bacteroidales*	*S24-7*	
**OTU0173**	*Bacteroidetes*	*Bacteroidia*	*Bacteroidales*	*S24-7*	
**OTU0036**	*Bacteroidetes*	*Bacteroidia*	*Bacteroidales*	*S24-7*	
**OTU0009**	*Bacteroidetes*	*Bacteroidia*	*Bacteroidales*	*S24-7*	
**OTU0010**	*Bacteroidetes*	*Bacteroidia*	*Bacteroidales*	*S24-7*	
**OTU0648**	*Firmicutes*	*Bacteroidia*	*Bacteroidales*	*Lachnospiraceae;*	*Clostridium*
**OTU0776**	*Firmicutes*	*Clostridia*	*Clostridiales*	*Lachnospiraceae*	*Clostridium*
**OTU0046**	*Firmicutes*	*Clostridia*	*Clostridiales*	*Lachnospiraceae*	*Roseburia*
**OTU0137**	*Firmicutes*	*Clostridia*	*Clostridiales*	*Lachnospiraceae*	*Ruminococcus*
**OTU0141**	*Firmicutes*	*Clostridia*	*Clostridiales*	*Ruminococcaceae*	*Ruminococcus*
**OTU0215**	*Firmicutes*	*Clostridia*	*Clostridiales*	*Lachnospiraceae*	*Ruminococcus*
**OTU0102**	*Firmicutes*	*Clostridia*	*Clostridiales*	*Ruminococcaceae*	*Ruminococcus*
**OTU0404**	*Proteobacteria*	*Gammaproteobacteria*	*Pseudomonadales*	*Moraxellaceae*	*Psychrobacter*
**OTU0028**	*Proteobacteria*	*Betaproteobacteria*	*Burkholderiales*	*Alcaligenaceae*	*Sutterella*
**OTU0914**	*Proteobacteria*	*Betaproteobacteria*	*Burkholderiales*	*Alcaligenaceae*	*Sutterella*
**OTU0348**	*Proteobacteria*	*Betaproteobacteria*	*Burkholderiales*	*Alcaligenaceae*	*Sutterella*
**OTU0225**	*Actinobacteria*	*Actinobacteria*	*Actinomycetales*	*Corynebacteriaceae*	*Corynebacterium*

Red: OTUs with enhanced abundance compared with that in the HFD group. Green: OTUs with reduced abundance compared with that in the HFD group.

**Table A2:** Fifty key OTUs responding to POP, as identified by redundancy analysis.

OTU ID	NC (%)	HFD (%)	HFD + SIM (%)	HFD + POP (%)
**OTU0012**	5.372650324	1.141401927	4.725000916	8.790443736
**OTU1365**	0.349016159	0.061375545	0.320618519	0.659558096
**OTU0005**	47.49459529	6.268550071	22.91139936	13.10230479
**OTU0911**	0.28947272	0.14473636	0.137407937	0.398483016
**OTU0994**	0.211608222	0.076948445	0.076948445	0.165805577
**OTU1497**	0.277564032	0.05679528	0.422300392	0.184126635
**OTU0024**	5.928694441	1.177127991	5.130812356	5.421201129
**OTU0241**	0.641237038	0.000005496	0.000916053	0.373749588
**OTU0030**	9.535194753	3.48008501	5.360741636	7.637133121
**OTU0051**	3.276721263	0.20153164	2.815946649	2.203107251
**OTU0056**	2.630903961	0.660474149	2.618995273	2.900223517
**OTU0080**	0.278480085	0.000005496	0.013740794	0.421384339
**OTU0105**	2.143563812	0.207944011	1.014070573	0.714521271
**OTU0170**	40.04800117	11.96639918	43.27250742	24.93679235
**OTU0003**	0.935290022	0.485508043	1.000329779	0.786889451
**OTU0359**	0.543219376	0.330695101	0.732842329	0.522150159
**OTU0998**	0.483675937	0.126415302	0.275731926	0.246418233
**OTU1044**	0.181378476	0.000005496	0.045802646	0.061375545
**OTU0194**	0.603678868	0.003664212	0.170385841	0.080612656
**OTU0283**	0.137407937	0.025649482	0.327946942	0.179546371
**OTU0295**	0.057711333	0.004580265	0.250082445	0.284892455
**OTU0391**	0.07236818	0.0000054963	0.025649482	0.146568466
**OTU0442**	0.184126635	0.10076582	0.205195852	0.215272434
**OTU0873**	0.517569895	0.000054963	0.280312191	0.282144297
**OTU0885**	0.691619948	0.341687736	1.119416658	0.753911546
**OTU0076**	0.360008794	0.159393207	3.259316258	0.124583196
**OTU0352**	0.174966106	0.010076582	0.12916346	0.11633872
**OTU0717**	0.124583196	0.000916053	0.029313693	0.005496317
**OTU0149**	0.603678868	0.222600857	0.377413799	0.331611154
**OTU0141**	0.956359239	0.000916053	0.000916053	0.447949874
**OTU0151**	0.000916053	0.198783482	0.07878055	0.020153164
**OTU0061**	0.240921916	2.157304606	0.580777546	0.473599355
**OTU0055**	0.000916053	5.28012898	0.002748159	1.152394562
**OTU0161**	0.402147228	0.560624382	0.298633249	0.272983768
**OTU0017**	4.220255762	10.22864681	5.834340992	4.274302884
**OTU0173**	0.250998498	0.575281228	0.296801143	0.137407937
**OTU0036**	1.261404859	10.95874098	1.946612436	0.84002052
**OTU0009**	4.281631307	11.87662599	2.474258913	5.92503023
**OTU0010**	5.066688652	9.421604192	4.130482577	3.033967242
**OTU0648**	0.261991133	2.5173134	1.014986626	0.085192921
**OTU0776**	0.003664212	0.030229746	0.014656847	0.010076582
**OTU0046**	0.112674508	16.8352204	0.555128064	0.272067715
**OTU0137**	0.0000054963	0.387490381	0.0000054963	0.001832106
**OTU0215**	0.000005.49632	0.22351691	0.070536074	0.043054487
**OTU0102**	0.000916053	0.551463853	0.246898245	0.263823238
**OTU0404**	0.02839764	0.294052985	0.030229746	0.053131069
**OTU0028**	2.104173537	13.70598366	1.250412224	1.134073504
**OTU0914**	0.058627386	0.455278297	0.032977905	0.030229746
**OTU0348**	0.02839764	0.162141365	0.040306328	0.023817376
**OTU0225**	0.110842402	0.838188414	0.199699535	0.21069217

Red: OTUs with enhanced abundance compared with that in the HFD group. Green: OTUs with reduced abundance compared with that in the HFD group.

**Table A3:** Primer sequences for amplification of target genes.

Target Gene ^1^	Primer Sequence (5′→3′)	Product Length, bp	Accession No. ^2^
*Ppara*	Forward (F): TGAAAGATTCGGAAACTGC	111	NM_013196
	Reverse (R): TGAAAGATTCGGAAACTGC		
*Pparg*	F: TCTCACAATGCCATCAGGTTTG	86	NM_013124
	R: AGCTGGTCGATATCACTGGAG		
*Fas*	F: ACCTCAGCAGCACATCTCAC	99	NM_017332
	R: CGTCCCTGTACACGTTCATC		
*Srebf1*	F: CGCTACCGTTCCTCTATCAATGAC	140	NM_001276707
	R: AGTTTCTGGTTGCTGTGCTGTAAG		
*Atgl*	F: ACCTGTG CCTTACCGTTCAC	144	NM_012859
	R: GGCAAGAGTGACAT GCAGAA		
*Fabp4*	F: ATGAAAGAAGTGGGAGTTGGC	139	NM_053365
	R: GTTTGAAGGAAATCTCGGTGTT		
*Cebpa*	F: CGTGGAGACGCAGCAGAAGGTGTT	127	NM_012524
	R: CAGAATCTCCTAGTCCTGGCTTGCA		
*Cebpb*	F: GGGTTTCGGGACTTGATGCA	270	NM_024125
	R: ATGCTCGAAACGGAAAAGGT		
β-actin	F: AAGGCCAACCGTGAAAAGAT	109	NM_031144
	R: TGGTACGACCAGAGGCATAC		

^1^ Ppara = peroxisome proliferator-activated receptor α; Pparg = peroxisome proliferator-activated receptor γ; Fas = fatty acid synthase; Srebf1 = sterol regulatory element-binding transcription factor 1; Atgl = adipose triglyceride lipase; Fabp4 = fatty acid binding protein 4; Cebpa = CCAAT/enhancer-binding protein alpha; Cebpb = CCAAT/enhancer-binding protein beta. ^2^ The reference sequence number is given for primers whose source is the National Center for Biotechnology Information (NCBI) GenBank database (http://www.ncbi.nlm.nih.gov/genbank/).

## References

[1] World Health Organization WHO-Obesity and Overweight Key Facts Sheet. http://www.who.int/en/news-room/fact-sheets/detail/obesity-andoverweight.

[2] Igel L.I., Saunders K.H., Fins J.J. (2018). Why weight? An analytic review of obesity management, diabetes prevention, and cardiovascular risk reduction. Curr. Atheroscler. Rep..

[3] Sun L., Ma L., Ma Y., Zhang F., Zhao C., Nie Y. (2018). Insights into the role of gut microbiota in obesity: Pathogenesis, mechanisms, and therapeutic perspectives. Protein Cell.

[4] Shahid S.U., Irfan U. (2018). The gut microbiota and its potential role in obesity. Future Microbiol..

[5] Damms-Machado A., Mitra S., Schollenberger A.E., Kramer K.M., Meile T., Konigsrainer A., Huson D.H., Bischoff S.C. (2015). Effects of surgical and dietary weight loss therapy for obesity on gut microbiota composition and nutrient absorption. BioMed Res. Int..

[6] Sen T., Cawthon C.R., Ihde B.T., Hajnal A., DiLorenzo P.M., de La Serre C.B., Czaja K. (2017). Diet-driven microbiota dysbiosis is associated with vagal remodeling and obesity. Physiol. Behav..

[7] Zeng H., Ishaq S.L., Zhao F.Q., Wright A.G. (2016). Colonic inflammation accompanies an increase of beta-catenin signaling and lachnospiraceae/streptococcaceae bacteria in the hind gut of high-fat diet-fed mice. J. Nutr. Biochem..

[8] Cui C., Li Y., Gao H., Zhang H., Han J., Zhang D., Li Y., Zhou J., Lu C., Su X. (2017). Modulation of the gut microbiota by the mixture of fish oil and krill oil in high-fat diet-induced obesity mice. PLoS ONE.

[9] Singh D.P., Singh J., Boparai R.K., Zhu J., Mantri S., Khare P., Khardori R., Kondepudi K.K., Chopra K., Bishnoi M. (2017). Isomalto-oligosaccharides, a prebiotic, functionally augment green tea effects against high fat diet-induced metabolic alterations via preventing gut dysbacteriosis in mice. Pharmacol. Res..

[10] Schroeder B.O., Backhed F. (2016). Signals from the gut microbiota to distant organs in physiology and disease. Nat. Med..

[11] Shi L.L., Li Y., Wang Y., Feng Y. (2015). Mdg-1, an ophiopogon polysaccharide, regulate gut microbiota in high-fat diet-induced obese c57bl/6 mice. Int. J. Biol. Macromol..

[12] Zhang X., Zhao Y., Xu J., Xue Z., Zhang M., Pang X., Zhang X., Zhao L. (2015). Modulation of gut microbiota by berberine and metformin during the treatment of high-fat diet-induced obesity in rats. Sci. Rep..

[13] Xu X., Xu P., Ma C., Tang J., Zhang X. (2013). Gut microbiota, host health, and polysaccharides. Biotechnol. Adv..

[14] Chen G., Xie M., Wan P., Chen D., Dai Z., Ye H., Hu B., Zeng X., Liu Z. (2018). Fuzhuan brick tea polysaccharides attenuate metabolic syndrome in high-fat diet induced mice in association with modulation in the gut microbiota. J. Agric. Food Chem..

[15] Lu J.M., Wang Y.F., Yan H.L., Lin P., Gu W., Yu J. (2016). Antidiabetic effect of total saponins from polygonatum kingianum in streptozotocin-induced daibetic rats. J. Ethnopharmacol..

[16] Li C., Chen J., Lu B., Shi Z., Wang H., Zhang B., Zhao K., Qi W., Bao J., Wang Y. (2014). Molecular switch role of akt in *Polygonatum odoratum* lectin-induced apoptosis and autophagy in human non-small cell lung cancer a549 cells. PLoS ONE.

[17] Wang Y., Qin S., Pen G., Chen D., Han C., Miao C., Lu B., Su C., Feng S., Li W. (2017). Original research: Potential ocular protection and dynamic observation of polygonatum sibiricum polysaccharide against streptozocin-induced diabetic rats’ model. Exp. Biol. Med..

[18] Zhao P., Zhao C., Li X., Gao Q., Huang L., Xiao P., Gao W. (2018). The genus polygonatum: A review of ethnopharmacology, phytochemistry and pharmacology. J. Ethnopharmacol..

[19] Gu M., Zhang Y., Fan S., Ding X., Ji G., Huang C. (2013). Extracts of rhizoma polygonati odorati prevent high-fat diet-induced metabolic disorders in c57bl/6 mice. PLoS ONE.

[20] Jiang Q., Lv Y., Dai W., Miao X., Zhong D. (2013). Extraction and bioactivity of polygonatum polysaccharides. Int. J. Biol. Macromol..

[21] Chen Y., Yin L., Zhang X., Wang Y., Chen Q., Jin C., Hu Y., Wang J. (2014). Optimization of alkaline extraction and bioactivities of polysaccharides from rhizome of *Polygonatum odoratum*. BioMed Res. Int..

[22] Yan H., Lu J., Wang Y., Gu W., Yang X., Yu J. (2017). Intake of total saponins and polysaccharides from polygonatum kingianum affects the gut microbiota in diabetic rats. Phytomedicine.

[23] Shu X.S., Lv J.H., Tao J., Li G.M., Li H.D., Ma N. (2009). Antihyperglycemic effects of total flavonoids from *Polygonatum odoratum* in stz and alloxan-induced diabetic rats. J. Ethnopharmacol..

[24] De Filippo C., Cavalieri D., Di Paola M., Ramazzotti M., Poullet J.B., Massart S., Collini S., Pieraccini G., Lionetti P. (2010). Impact of diet in shaping gut microbiota revealed by a comparative study in children from Europe and rural Africa. Proc. Natl. Acad. Sci. USA.

[25] Turnbaugh P.J., Ley R.E., Mahowald M.A., Magrini V., Mardis E.R., Gordon J.I. (2006). An obesity-associated gut microbiome with increased capacity for energy harvest. Nature.

[26] Zhou S.S., Xu J., Zhu H., Wu J., Xu J.D., Yan R., Li X.Y., Liu H.H., Duan S.M., Wang Z. (2016). Gut microbiota-involved mechanisms in enhancing systemic exposure of ginsenosides by coexisting polysaccharides in ginseng decoction. Sci. Rep..

[27] Foldy C.J., Dyhrfjeld-Johnsen J., Soltesz I. (2005). Structure of cortical microcircuit theory. J. Physiol..

[28] Zhang H., DiBaise J.K., Zuccolo A., Kudrna D., Braidotti M., Yu Y., Parameswaran P., Crowell M.D., Wing R., Rittmann B.E. (2009). Human gut microbiota in obesity and after gastric bypass. Proc. Natl. Acad. Sci. USA.

[29] Kobayashi T., Osaki T., Oikawa S. (2015). Use of t-rflp and seven restriction enzymes to compare the faecal microbiota of obese and lean Japanese healthy men. Benef. Microbes.

[30] Handl S., German A.J., Holden S.L., Dowd S.E., Steiner J.M., Heilmann R.M., Grant R.W., Swanson K.S., Suchodolski J.S. (2013). Faecal microbiota in lean and obese dogs. FEMS Microbiol. Ecol..

[31] Li H., Qi T., Huang Z.S., Ying Y., Zhang Y., Wang B., Ye L., Zhang B., Chen D.L., Chen J. (2017). Relationship between gut microbiota and type 2 diabetic erectile dysfunction in sprague-dawley rats. J. Huazhong Univ. Sci. Technol. Med. Sci..

[32] Song J.J., Tian W.J., Kwok L.Y., Wang Y.L., Shang Y.N., Menghe B., Wang J.G. (2017). Effects of microencapsulated lactobacillus plantarum lip-1 on the gut microbiota of hyperlipidaemic rats. Br. J. Nutr..

[33] Tang C., Sun J., Zhou B., Jin C., Liu J., Kan J., Qian C., Zhang N. (2018). Effects of polysaccharides from purple sweet potatoes on immune response and gut microbiota composition in normal and cyclophosphamide treated mice. Food Funct..

[34] Mach N., Moisan G.P., Foury A., Kittelmann S., Reigner F., Moroldo M., Ballester M., Esquerré D., Riviere J., Salle G. (2017). The effects of weaning methods on gut microbiota composition and horse physiology. Front. Physiol..

[35] He M., Fang S., Huang X., Zhao Y., Ke S., Yang H., Li Z., Gao J., Chen C., Huang L. (2016). Evaluating the contribution of gut microbiota to the variation of porcine fatness with the cecum and fecal samples. Front. Microbiol..

[36] Den Besten G., Lange K., Havinga R., van Dijk T.H., Gerding A., van Eunen K., Muller M., Groen A.K., Hooiveld G.J., Bakker B.M. (2013). Gut-derived short-chain fatty acids are vividly assimilated into host carbohydrates and lipids. Am. J. Physiol. Gastrointest. Liver Physiol..

[37] Zhou W., Yan Y., Mi J., Zhang H., Lu L., Luo Q., Li X., Zeng X., Cao Y. (2018). Simulated digestion and fermentation in vitro by human gut microbiota of polysaccharides from bee collected pollen of Chinese wolfberry. J. Agric. Food Chem..

[38] Miller P.G., Bonn M.B., Franklin C.L., Ericsson A.C., McKarns S.C. (2015). TNFR2 deficiency acts in concert with gut microbiota to precipitate spontaneous sex-biased central nervous system demyelinating autoimmune disease. J. Immunol..

[39] Williams O.M., Brazier J., Peraino V., Goldstein E.J. (2010). A review of three cases of *Clostridium aldenense* bacteremia. Anaerobe.

[40] Salonen A., de Vos W.M. (2014). Impact of diet on human intestinal microbiota and health. Annu. Rev. Food Sci. Technol..

[41] Yang H., Xiao Y., Gui G., Li J., Wang J., Li D. (2018). Microbial community and short-chain fatty acid profile in gastrointestinal tract of goose. Poult. Sci..

[42] Yang H., Xiao Y., Wang J., Xiang Y., Gong Y., Wen X., Li D. (2018). Core gut microbiota in jinhua pigs and its correlation with strain, farm and weaning age. J. Microbiol..

[43] Wang X., Wang X., Jiang H., Cai C., Li G., Hao J., Yu G. (2018). Marine polysaccharides attenuate metabolic syndrome by fermentation products and altering gut microbiota: An overview. Carbohydr. Polym..

[44] Nihei N., Okamoto H., Furune T., Ikuta N., Sasaki K., Rimbach G., Yoshikawa Y., Terao K. (2018). Dietary Alpha-Cyclodextrin Modifies Gut Microbiota and Reduces Fat Accumulation in High-Fat-Diet-Fed Obese Mice.

[45] Cho K.W., Kim Y.O., Andrade J.E., Burgess J.R., Kim Y.C. (2011). Dietary naringenin increases hepatic peroxisome proliferators-activated receptor alpha protein expression and decreases plasma triglyceride and adiposity in rats. Eur. J. Nutr..

[46] Shimano H. (2009). Srebps: Physiology and pathophysiology of the SREBP family. FEBS J..

[47] Camargo A., Meneses M.E., Perez-Martinez P., Delgado-Lista J., Rangel-Zuniga O.A., Marin C., Almaden Y., Yubero-Serrano E.M., Gonzalez-Guardia L., Fuentes F. (2014). Dietary fat modifies lipid metabolism in the adipose tissue of metabolic syndrome patients. Genes Nutr..

[48] Lee M., Sung S.H. (2016). Platyphylloside isolated from *Betula platyphylla* inhibit adipocyte differentiation and induce lipolysis via regulating adipokines including ppargamma in 3t3-l1 cells. Pharmacogn. Mag..

[49] Okuno A., Tamemoto H., Tobe K., Ueki K., Mori Y., Iwamoto Y., Umesono K., Akanuma Y., Fujiwara T., Horikoshi H. (1998). Troglitazone increases the number of small adipocytes without the change of white adipose tissue mass in obese zucker rats. J. Clin. Investig..

[50] Kota B.P., Huang T.H., Roufogalis B.D. (2005). An overview on biological mechanisms of ppars. Pharmacol. Res..

[51] Garin-Shkolnik T., Rudich A., Hotamisligil G.S., Rubinstein M. (2014). Fabp4 attenuates ppargamma and adipogenesis and is inversely correlated with ppargamma in adipose tissues. Diabetes.

[52] Odegaard J.I., Ricardo-Gonzalez R.R., Goforth M.H., Morel C.R., Subramanian V., Mukundan L., Red Eagle A., Vats D., Brombacher F., Ferrante A.W. (2007). Macrophage-specific ppargamma controls alternative activation and improves insulin resistance. Nature.

[53] Heming M., Gran S., Jauch S.L., Fischer-Riepe L., Russo A., Klotz L., Hermann S., Schafers M., Roth J., Barczyk-Kahlert K. (2018). Peroxisome proliferator-activated receptor-gamma modulates the response of macrophages to lipopolysaccharide and glucocorticoids. Front. Immunol..

[54] Guo Y.R., Choung S.Y. (2017). Germacrone attenuates hyperlipidemia and improves lipid metabolism in high-fat diet-induced obese c57bl/6j mice. J. Med. Food.

[55] Qiao Y., Sun J., Xia S., Tang X., Shi Y., Le G. (2014). Effects of resveratrol on gut microbiota and fat storage in a mouse model with high-fat-induced obesity. Food Funct..

[56] Yan H., Ajuwon K.M. (2015). Mechanism of butyrate stimulation of triglyceride storage and adipokine expression during adipogenic differentiation of porcine stromovascular cells. PLoS ONE.

[57] Jia Y., Hong J., Li H., Hu Y., Jia L., Cai D., Zhao R. (2017). Butyrate stimulates adipose lipolysis and mitochondrial oxidative phosphorylation through histone hyperacetylation-associated beta3-adrenergic receptor activation in high-fat diet-induced obese mice. Exp. Physiol..

[58] Chandler P.C., Viana J.B., Oswald K.D., Wauford P.K., Boggiano M.M. (2005). Feeding response to melanocortin agonist predicts preference for and obesity from a high-fat diet. Physiol. Behav..

[59] Cheng H., Xu N., Zhao W., Su J., Liang M., Xie Z., Wu X., Li Q. (2017). (−)-Epicatechin regulates blood lipids and attenuates hepatic steatosis in rats fed high-fat diet. Mol. Nutr. Food Res..

[60] Liu N., Dong Z., Zhu X., Xu H., Zhao Z. (2018). Characterization and protective effect of polygonatum sibiricum polysaccharide against cyclophosphamide-induced immunosuppression in balb/c mice. Int. J. Biol. Macromol..

[61] Pourabedin M., Guan L., Zhao X. (2015). Xylo-oligosaccharides and virginiamycin differentially modulate gut microbial composition in chickens. Microbiome.

[62] Fadrosh D.W., Ma B., Gajer P., Sengamalay N., Ott S., Brotman R.M., Ravel J. (2014). An improved dual-indexing approach for multiplexed 16S rRNA gene sequencing on the Illumina MiSeq platform. Microbiome.

[63] Cole J.R., Wang Q., Fish J.A., Chai B., McGarrell D.M., Sun Y., Brown C.T., Porras-Alfaro A., Kuske C.R., Tiedje J.M. (2014). Ribosomal database project: Data and tools for high throughput rrna analysis. Nucleic Acids Res..

[64] Edgar R.C. (2013). UPARSE: Highly accurate OTU sequences from microbial amplicon reads. Nat. Methods.

